# Camel-Derived Nanobodies as Potent Inhibitors of New Delhi Metallo-β-Lactamase-1 Enzyme

**DOI:** 10.3390/molecules29071431

**Published:** 2024-03-22

**Authors:** Rahma Ben Abderrazek, Emna Hamdi, Alessandra Piccirilli, Sayda Dhaouadi, Serge Muyldermans, Mariagrazia Perilli, Balkiss Bouhaouala-Zahar

**Affiliations:** 1 Laboratoire des Biomolécules Venins et Applications Théranostiques, Institut Pasteur Tunis, 13 Place Pasteur, Tunisie Université Tunis El Manar, B.P N 93, Tunis 1068, Tunisia; emna.hamdi@etudiant-fst.utm.tn (E.H.); sayda.dhaouadi@pasteur.utm.tn (S.D.); balkiss.bouhaouala@fmt.utm.tn (B.B.-Z.); 2 Dipartimento di Scienze Cliniche Applicate e Biotecnologiche, Università degli Studi dell’Aquila, Via Veteoio Coppito, 67100 L’Aquila, Italy; alesssandra.piccirilli@univaq.it (A.P.); mariagrazia.perilli@univaq.it (M.P.); 3 Laboratory of Cellular and Molecular Immunology, Vrije Universiteit Brussel, Pleenlaan, 9, 1050 Brussels, Belgium; serge.muyldermans@vub.be; 4 Faculté de Médecine de Tunis, Université Tunis El Manar, B.P N 93, Tunis 1068, Tunisia

**Keywords:** phage display, nanobodies, NDM-1, metallo-β-lactamases, β-lactamase small molecule inhibitors

## Abstract

The injudicious usage of antibiotics during infections caused by Gram-negative bacteria leads to the emergence of β-lactamases. Among them, the NDM-1 enzyme poses a serious threat to human health. Developing new antibiotics or inhibiting β-lactamases might become essential to reduce and prevent bacterial infections. Nanobodies (Nbs), the smallest antigen-binding single-domain fragments derived from Camelidae heavy-chain-only antibodies, targeting enzymes, are innovative alternatives to develop effective inhibitors. The biopanning of an immune VHH library after phage display has helped to retrieve recombinant antibody fragments with high inhibitory activity against recombinant-NDM-1 enzyme. Nb02NDM-1, Nb12NDM-1, and Nb17NDM-1 behaved as uncompetitive inhibitors against NDM-1 with K_i_ values in the nM range. Remarkably, *IC*_50_ values of 25.0 nM and 8.5 nM were noted for Nb02NDM-1 and Nb17NDM-1, respectively. The promising inhibition of NDM-1 by Nbs highlights their potential application in combating particular Gram-negative infections.

## 1. Introduction

β-lactams are the most significant and frequently used antibiotics in clinical practice [1]. Nevertheless, because of their widespread clinical use and even misuse, most pathogens have developed various resistance mechanisms against this class of antibiotics [2,3,4].

The main mechanism of resistance to β-lactams is the production of β-lactamases. These well-studied enzymes account for more than 7000 variants [5]. Currently, metallo-β-lactamases (MβLs) are widely disseminated, representing a serious global public health problem [6]. Based on their amino acid sequences and catalytic properties, MβLs are classified into three major structural subclasses (B1, B2, and B3) [7]. The MβLs belonging to subclass B1, such as IMP-, VIM-, and NDM-variants, are widely diffused in Enterobacterales, specifically *Klebsiella pneumoniae*, *Escherichia coli*, and *Enterobacter cloacae,* causing severe hospital-acquired infections [8,9,10,11,12].

The increasing number of MβL inhibitors is a response to the rising prevalence of the carbapenem-resistant bacterial phenotype in clinical settings. Noteworthily, clinical inhibitors approved for MβLs are not available, and first-generation β-lactamase inhibitors, such as tazobactam, sulbactam, and clavulanic acid, are ineffective against MβLs [13,14]. In addition, some boronate compounds (i.e., Taniborbactam or QPX7728) seem to possess a good inhibitory activity against MβLs (not including IMP-1 enzyme), but they are not yet approved by drug regulatory authorities [15,16,17].

In this context, the development of new antibodies acting as enzyme inhibitors seems to be an attractive strategy for addressing the ongoing complexities associated with antibiotic resistance. While the development of single lactamase inhibitors may not completely resolve it, they could still contribute significantly by reducing its impact [18].

The variable domains of heavy-chain-only antibodies derived from camelid, referred to as Nbs, are among the smallest (15 kDa), intact, antigen-binding units [19]. They target specific epitopes that are less preferred or even inaccessible to conventional antibodies, such as the clefts of an enzyme’s active site. Hence, they have proven to be a much better option for obtaining small inhibitory molecules against MβL enzymes [20].

In our previous study, we clearly demonstrated the inhibitory potential of NDM-1-specific IgGs purified from dromedary serum immunized with this antigen [21]. Following this proof of concept, the present challenge is to design a practical strategy to identify Nbs with a high inhibitory potency toward the NDM-1 enzyme.

Therefore, the generation of Nbs using phage display technology against recombinant NDM-1 enzyme has been successfully reported. The amounts of Nb02NDM-1, Nb12NDM-1, and Nb17NDM-1 expressed in *E. coli* were estimated and used for functional characterization. Their ability to recognize and inhibit NDM-1 was confirmed using both ELISA and kinetic inhibition assays.

## 2. Results and Discussion

### 2.1. Library Construction

An immune response against NDM-1 recombinant enzyme has been raised in the immunized dromedary as previously described [21]. Extracted cDNA was used as a template in the first PCR using CALL001/CALL002-specific primers. As expected, two PCR fragments were noted after agarose gel electrophoresis: 700 and 900 bp fragments (Supplementary Figure S1A). The VHH fragments were selectively amplified in a nested PCR using primers specifically annealing in Framework-Region1 and Framework-Region 4 (Supplementary Figure S1B) [22]. The VHH-library in pMECS transformed in TG1 cells is estimated to contain 3.4 × 10^7^ CFU/mL, emphasizing the substantial diversity of the library and potentially providing a rich repertoire of nanobodies for subsequent screening and selection. After colony PCR, more than 90% of the randomly selected, i.e., 32 clones, were shown to contain an insert with the correct size for the VHH gene (Supplementary Figure S1C). Such a library displays a good starting point to retrieve Nbs with a high affinity and specificity to the NDM-1 immunogen. To increase the likelihood of obtaining high-specificity binders to the cognate target antigen, a good immune Nb library should contain >10^7^ individual transformants and have a VHH insertion rate of at least 70% [23,24].

### 2.2. Selection of NDM-1-Specific VHH Fragments after Phage Display and Biopanning

**Figure 1:**
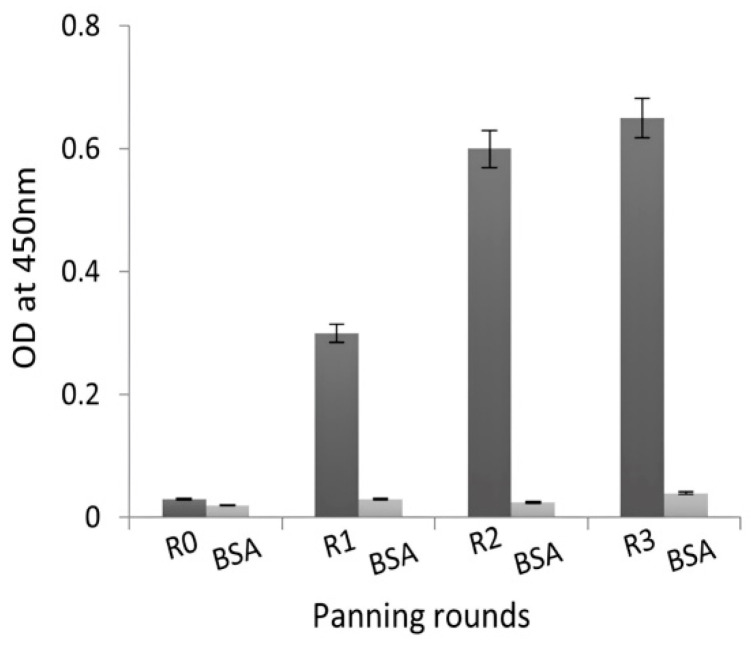
Evaluation of phage particle enrichment with target-specific Nbs during sequential biopanning steps using polyclonal phage ELISA. A total of 10^10^ phages from each round of panning were evaluated for their binding activity against the NDM-1 enzyme. BSA was used as a negative control. R0 represents the unpanned library, and R1–R3 represents VHH-virions eluted after rounds 1–3 of library panning. Values are the average of two replicate wells, and error bars designate the standard deviation.

An aliquot of the library covering a representative sample of the recombinant VHH bank was phage-displayed after being rescued by M13KO7 helper phages. The three rounds of panning on the immobilized NDM-1 enzyme enriched phage particles expressing antigen-specific Nbs. The number of input phages was 10^12^ in the first round and 10^9^ in the second and third rounds. As expected, the consecutive rounds of selection gradually increase the fraction of NDM-1-specific phages [25]. In addition, the dual use of purified NDM-1 and increasing stringency during the consecutive in vitro washings directed the likelihood of selecting target-specific binders. Specifically, the number of washes increased from 10 in the first round, to 15 in the second round, and 20 in the third round. The enrichment of clones expressing antigen-specific Nbs during second and third rounds of biopanning is illustrated by the results of a polyclonal phage ELISA performed on phages eluted after each round of biopanning (Figure 1).

**Figure 2:**
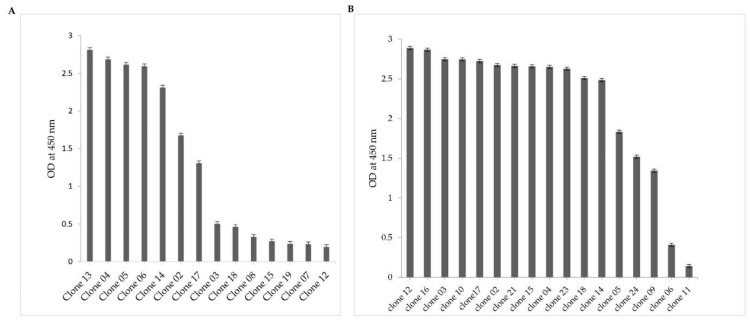
Selection of NDM-1-specific VHH fragments. The periplasmic products of randomly picked clones were tested using ELISA. Clones with at least 2× stronger ELISA signals on NDM-1-coated wells vs. non-coated wells were considered positive. (**A**) Selection of NDM-1-specific VHH fragments after the second round of panning. (**B**) Selection of NDM-1-specific VHH fragments after the third round of panning.

Forty-eight individual colonies from second and third rounds of panning were randomly selected, and VHH expression was induced with IPTG, leading to the accumulation of Nbs in the bacterial periplasm. Periplasmic extracts obtained from individual colonies were then tested using ELISA to detect NDM-1-specific binders. Out of 48 clones, 17 extracts did not yield a significant signal. Interestingly, 31 extracts exhibited a strong signal in ELISA, reaching at least 4× the background signal and reflecting a combination of high affinity and/or a good production yield (background signal from 0.02 to 0.5 OD/mL) (Figure 2A,B). Each produced extract was tested in duplicate wells.

### 2.3. VHH Sequence Analysis

**Figure 3:**
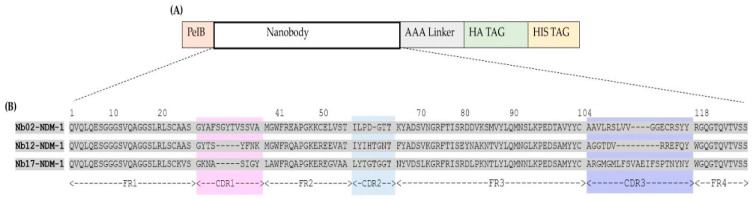
(**A**) Schematic illustration of Nb in the phagemid vector pMECS. Nb sequence was tracked by a triple alanine linker, hemagglutinin (HA), and hexa-histidine (His) tags. (**B**) Amino acid sequences of NDM-1-specific Nbs, numbered according to IMGT [26]. CDR1, CDR2, and CDR3 are indicated in pink, blue, and purple, respectively. The Framework regions (FR) and hypervariable regions or complementarity determining regions (CDR) are indicated at the bottom of the figure.

The nanobody sequences of 31 clones that scored positive in ELISA were aligned (Figure 3) and numbered according to ImMunoGeneTics numbering (IMGT) [26] and grouped into three distinct clusters based on the homology of their CDR3, indicating that there are three unique sequences among the 31 duplicate clones. Notably, each of these unique sequences was approximately represented 10 times. These Nbs are referred to as Nb02NDM-1 (Figure 2A), Nb12NDM-1, and Nb17NDM-1 (Figure 2B). It has been reported that the CDR3 of Nbs constitutes 60–80% of the antigen contacting area [27], and that a synthetic peptide derived from CDR3 (peptibody) preserves measurable affinity and specificity to antigen [28]. Furthermore, the three selected Nbs originated from an independent B-cell lineage, as they share a low degree of sequence identity and have a different CDR3 length. The length of the CDR3 is from 12 to 21 amino acids among the selected Nbs, and Nb17NDM-01 has the longest CDR3 of all.

Nb12NDM-1 and Nb17NDM-1 contain VHH hallmark amino acids in their Framework-Region 2. Remarkably, the sequence analysis of Nb02NDM-1 reveals the presence of an extra pair of Cys residues apart from the conventional Cys23 and Cys104 with a Cys residue at position 50 and an extra Cys residue at the end of CDR3, which is frequently observed for dromedary VHHs [29,30]. More remarkably, Nb02NDM-1 possesses an intermediate CDR3 length and a longer CDR1 loop (13AA). Usually, the CDR1 has a length of eight residues [31] (Figure 3B).

### 2.4. Production and Purification of Nbs

Recombinant plasmid constructs were introduced by transformation into *E. coli* WK6 electrocompetent cells. The Nb02NDM-1, Nb12NDM-1, and Nb17NDM-1 were extracted from 1 L of culture in TB (Terrific Broth) and purified with two chromatographic steps. The average yield of purified Nb02NDM-1 is estimated to be 1.3 mg/L, whereas for Nb12NDM-1 and Nb17NDM-1, it is estimated to be 0.40 mg/L. The electrophoretic profiles of the Nb02NDM-1, Nb12NDM-1, and Nb17NDM-1 purification products showed the presence of a 15 kDa molecular weight apparent band (Supplementary Figure S2, lanes 1, 4, and 5). This is a surprisingly low level of expression for Nbs into the bacterial periplasm; thus, the fermentation of Nbs in yeasts or Gram-positive bacteria (i.e., *Baccillus subtilis*) might be considered in the future to increase the production level with correctly folded Nbs [32,33].

**Figure 4:**
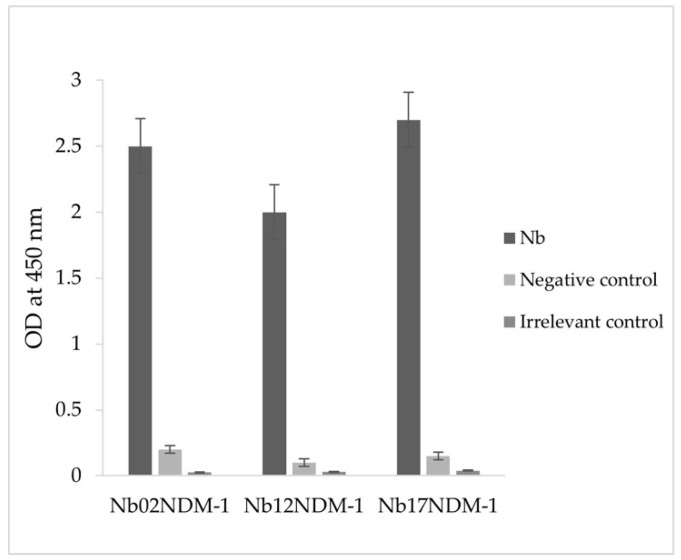
Reactivity of purified Nbs against NDM-1. The binding ability of purified Nbs using 1 µg/mL of recombinant NDM-1 enzyme was tested using ELISA. The three Nbs showed a strong signal at least 10-fold higher compared to the background signal, to assess non-specific binding, and compared to the irrelevant control signal, to demonstrate the specificity of binding with the antigen of interest. (Error bars indicating the standard deviation represent the variability observed between biological replicates).

An ELISA was then carried out to evaluate the binding capacity between 5 × 10^−3^ µg/mL of the purified Nbs and 1 µg/mL of the NDM-1 enzyme. As shown in Figure 4, all three Nbs showed positive interactions with the recombinant enzyme. Indeed, the absorbance values at OD_450 nm_ are at least more than 10-fold higher than those of both the negative controls (OD not exceeding 0.2) and the irrelevant control (scorpion toxin BotI), indicating the strong binding and specificity between Nbs and NDM-1. Interestingly, Nb12NDM-1 was slightly less effective to bind NDM-1 at equilibrium, compared to Nb02NDM-1 and Nb17NDM-1.

### 2.5. Inhibition Kinetic Assays

The inhibitory capacity of purified Nbs on recombinant NDM-1 was monitored from the initial hydrolysis rate of meropenem using a mixture of enzyme and Nb-based inhibitor. Under our experimental conditions, all three Nbs behaved as reversible inhibitors in an uncompetitive manner. Indeed, the K_m_ and k_cat_ values decreased as the Nbs concentration increased (Table 1).

As shown in Table 2, Nb02NDM-1, Nb12NDM-1, and Nb17NDM-1 inhibit NDM-1 with K_i_ values of 4.7 nM, 117 nM, and 3.7 nM, respectively. The experimental *IC*_50_ are 26.0 nM, 485 nM, and 7.6 nM for Nb02NDM-1, Nb12NDM-1, and Nb17NDM-1, respectively. The lowest *IC*_50_ value is recorded for Nb17NDM-1 (Table 2) (Supplementary Figure S3). Remarkably, Nb17NDM-1 has the longest CDR3 of all retrieved anti-NDM-1 binders (21 amino acids). It is well known that the lack of the light chain observed in dromedary heavy-chain-only antibodies is usually compensated by a longer CDR3 in the VHH compared to that of a VH. The paratope of conventional antibodies tends to form a flat surface or a groove [24]. In contrast, the paratope of VHHs has often a convex shape that prefers to interact with concave-shaped epitopes, such as the catalytic site of enzymes [34,35].

Therefore, the peptidomimetics of CDR3 of the “best in class” Nbs with high inhibitory capacity might be designed in the future to reach a low molecular weight for anti-NDM-1 inhibitory drugs. Indeed, it has been well demonstrated with crystallography that VHH can use only a single CDR loop to bind to the antigen with nanomolar affinity [36]. Further investigations will be conducted to comprehensively characterize these peptides, including their structure, function, specificity, and affinity towards the target NDM-1 molecule.

## 3. Materials and Methods

### 3.1. Enzyme, Vector, and Strains

NDM-1 metallo-β-lactamase was purified from an *E. coli* pET-24/NDM-1 culture [37]. To express a Nb tagged with a hemagglutinin (HA)-His6 tag at the C-terminus, a pMECS phagemid vector was used [38]. Initially, this plasmid was introduced into the amber-suppressed *E. coli* TG1 strain, resulting in the expression of the VHH–gene III fusion, which was then transferred to the non-suppressor WK6 strain to express only the VHH domains [39].

### 3.2. Library Construction

First, a female dromedary (*Camelus dromedarius*) is hyper-immunized to produce NDM-1-specific HCAbs as previously described [21]. After immunization, the lymphocytes were isolated from the collected blood samples. Total RNA was extracted, purified, and reverse transcribed into cDNA using SuperScript II First-Strand Synthesis System (catalog number: 18064-014, Invitrogen, Carlsbad, CA, USA). CALL001 (5′-GTCCTGGCTGCTCTTCTACAAGG-3′) and CALL002 (5′-GGTACGTGCTGTTGAACTGTTCC-3′) primers were used to amplify the variable domains of heavy-chain IgGs (VH-CH1-Hinge-CH2 of IgG1 and the VHH-Hinge-CH2 exons of IgG2 and IgG3). The resulting DNA fragment of approximately 700 bp was then extracted from the agarose gel using the Qiaquick Gel Extraction Kit (catalog number: 28704, Qiagen, Hilden, Germany). Subsequently, using a nested PCR, employing A6E (5′-GATGTGCAGCTGCAGGAGTCTGGRGGAGG-3′) and PMCF (5′-CTAGTGCGGCCGCTGAGGAGACGGTGACCTGGGT-3′) specific primers, the VHH gene fragment was amplified, and PstI and NotI restriction sites were inserted, respectively. The generated PCR amplicon was subjected to PstI and NotI restriction enzyme digestion (catalog numbers: R3140T and R3189M, New England Biolabs, Hitchin, UK, respectively), before ligating into the pMECS vector using T4 DNA Ligase (catalog number 15224-041 Invitrogen, Carlsbad, CA, USA). Ligation product was then used to transform freshly prepared *E. coli* TG1 electro-competent cells. After electroporation, 50 μL of recombinant cells was mixed with 1 mL of LB medium and plated on a selective medium. After overnight incubation at 37 °C, the colonies of TG1 cells are scraped from the large plates and stored in LB medium with 20% glycerol in 1 mL aliquots and stored at −80 °C. The percentage of clones carrying a phagemid with the appropriately sized VHH gene insert was evaluated using colony PCR on 48 randomly picked colonies using MP57 and GIII primers. The MP57 (5′-TTA TGC TTC CGG CTC GTA TG-3′) and GIII (5′-CCA CAG ACA GCC CTC ATA G-3′) primers bind to the vector sequences, flanking the cloned VHH insert [22].

### 3.3. Selection of NDM-1-Specific VHH Fragments Using Biopanning Phage Display

Specific VHH fragments targeting NDM-1 metallo-β-lactamase were enriched on microtiter plates coated with NDM-1 (10 μg/well) (catalog number: M5785-1CS; Sigma Aldrich, Saint Louis, MO, USA). A representative aliquot of the VHH library was inoculated in 300 mL 2×Y and grown until the early-logarithmic phase before the bacteria were infected with 10^12^ M13KO7 helper phages (catalog number: 170-3578, New England BioLabs, Hitchin, UK), incubated for 30 min without shaking and then centrifuged at low speed to pellet the bacteria. The cell pellet was resuspended in fresh medium (containing 100 μg/mL ampicillin and 70 μg/mL kanamycin) and shaken overnight at 37 °C. The bacteria were pelleted using low-speed centrifugation, and the recombinant phage particles were precipitated in polyethylene glycol (PEG)/NaCl solution. The virions were collected, resuspended in 1 mL of sterile PBS, titrated, and used as input phages for the panning process. To enrich phage virions carrying NDM-1-specific VHHs, three consecutive rounds of panning were performed on 10 μg of immobilized enzyme in a well of a microtiter plate (catalog number: M5785-1CS; Sigma Aldrich, MO, USA). NDM-1-specific virions were eluted with 100 mM triethylamine (pH 10.0, catalog number: T0886; Sigma Aldrich, MO, USA) after each selection, and neutralized immediately with 1.0 M Tris-HCl (pH 8.0; catalog number: CE234 Gene ON, Ludwigshafen, Germany). The phage particles were then used to infect exponentially growing *E. coli* TG1 strains. An aliquot was properly diluted and loaded onto LB Agar with ampicillin. The enrichment of phage particles carrying an enzyme-specific VHH was evaluated by comparing the number of virions eluted from wells coated with the NDM-1 recombinant enzyme versus wells without recombinant NDM-1. To evaluate the panning process, polyclonal phage ELISA was carried out using phage samples eluted after each round of panning (output phages). Antigen-specific bound phages were identified using an HRP-conjugated anti-M13 monoclonal antibody (catalog number: 27942101, Sigma Aldrich, MO, USA).

### 3.4. Screening for NDM-1-Specific Nbs

Individual colonies were randomly selected and cultivated in a 24-round-bottom well plate with 1 mL of 2×TY medium supplemented with 2% glucose and 100 µg/mL ampicillin. After 4–5 h of incubation, 1 mM IPTG was added to induce the expression of the encoded VHHs extended with HA and His tags. After overnight incubation, periplasmic proteins were extracted using osmotic shock using TES and TES/4 buffers (0.5 M sucrose, 0.2 M Tris-HCl pH 8, 0.5 mM EDTA), and each soluble extract was added to a well coated with 100 µL NDM-1 at 1 µg/mL and another well without the NDM-1 enzyme, which was used as a negative control. Antigen-bound Nbs were identified using rabbit anti-His IgG (catalog number: ab9108, Abcam, Waltham, MA, USA), which was diluted at a ratio of 1/5000, and goat anti-rabbit IgG conjugated with peroxidase (catalog number: ab6721, Abcam, USA) diluted at a ratio of 1/5000 [40]. Subsequently, colonies corresponding to the extracts that exhibited positive signals in ELISA (at least 2-fold higher than the background signal from a well without antigen) were cultured and used to extract phagemid DNA, from which the Nb insert was sequenced and analyzed. Following standard protocols, ELISA was conducted to assess the binding ability of purified Nbs to the NDM-1 enzyme. Microtiter plates (Nunc Maxisorp Plates, HTDS, Tunis, Tunisia) were coated with 100 µL of 1 µg/mL NDM-1 enzyme and 100 µL of 1 µg/mL BotG50 scorpion toxin used as irrelevant control in PBS buffer (Phosphate-Buffered Saline) and incubated overnight at 4 °C. After 5–6 successive washes with 0.1% Tween-PBS, the residual protein-binding sites were blocked by incubation with 5% skimmed milk in PBS for 1 h at 37 °C. After repeating the washing steps, 100 µL of 5 µg/mL Nbs (5 µg/mL) was added and incubated for 1 h at 37 °C. The formed antigen–Nbs complexes were detected using rabbit anti-His IgG (catalog number: ab9108, Abcam, USA) and goat anti-rabbit IgG conjugated with peroxidase (catalog number: ab6721, Abcam, USA), both of which were used at the same dilution (1/5000). Wells without the NDM-1 enzyme were used as negative controls. The optical density was measured at 450 nm using a spectrophotometer (Multiskan EX, Thermo Electron Corporation, Waltham, MA, USA).

### 3.5. VHH Sequence Analysis

The phagemid DNA extracted from clones that scored positive in the periplasmic extract-ELISA was sequenced (ABI prism 3100 Genetic Analyzer, Applied Biosystems, Waltham, MA, USA). Nucleotide sequences were translated into amino acid sequences, aligned according to IMGT unique numbering [26] and grouped into distinct clusters according to the length and sequence identity within the CDR3.

### 3.6. Nb Production and Purification

The pMECS phagemids encoding the VHH genes from ELISA-positive clones were isolated from *E. coli* TG1 cells, electroporated into *E. coli* WK6 cells, and cultured on selective agar plates. Individual colonies were picked and cultured for the expression of Nbs fused with HA and His tags at their C-terminal end. The transformed bacteria were shaken overnight at 37 °C in flasks containing Terrific Broth medium (TB, catalog number: 6247, Biotec, GR, Fermo, Italy) containing 100 µg/mL ampicillin and 0.1% glucose. The expression of Nbs was induced by the addition of IPTG (1 mM). After 16 h of incubation at 28 °C, bacterial cell pellets were treated with TES and TES/4 buffers (pH 7.0) to liberate periplasmic proteins [41]. The cleared lysate was dialyzed using a dialysis membrane (VISKING^®^ dialysis tubing 44114.04 SERVA, Heidelberg, Germany) O/N at 4 °C using PB buffer (NaH_2_PO_4_, Na_2_HPO_4_, 50 mM) and loaded onto a Sepharose-S fast-flow column (2.0 20 cm; GHealthcare, Milan, Italy) pre-equilibrated with a buffered solution depending on the isoelectric point (pI) of each Nb: sodium phosphate buffer (50 mM, pH 6.2) for the purification of Nb02NDM-1 and Nb17NDM-1, and acetate buffer (50 mM, pH 5.2) for Nb12NDM-1. Nb was eluted with a linear gradient of NaCl (0–1 M) in the same buffer. Fractions containing Nbs were pooled, concentrated 20-fold with an Amicon concentrator (Amicon^®^ Ultra-15 Centrifugal Filters Merck, YM 3 membrane, Merck, Tullagreen, Carrigtwohill, Co Cork IRL), and loaded onto a Superdex-G75 gel-filtration chromatography column (10/30, Pharmacia) pre-equilibrated with sodium phosphate buffer (pH 7.0) supplemented with 0.15 M NaCl. The eluted fractions were concentrated and verified using SDS–PAGE (16% gel). Nb concentrations were measured at 595 nm using the Bradford assay kit (catalog number: 500-0006 BIO-RAD, Munich, Germany).

### 3.7. Inhibition Kinetic Assays

To evaluate the inhibition capacity of the produced Nbs towards the NDM-1 enzyme, kinetic assays were performed using a Perkin-Elmer Lambda 25 spectrophotometer. Competitive inhibition assays with Nbs were performed using 100 μM meropenem $\left(∆\begin{array}{c} M\\ \varepsilon \end{array}297\;nm=-6500\;M^{-1}{cm}^{-1}\right)$ as a reporter substrate and 8 nM NDM-1. Measurements were performed in a 30 mM HEPES buffer (pH 7.0) containing 50 μM ZnCl_2_. K_i_ values were determined through the application of the subsequent equation, i.e., v_0_/v_i_ = 1 + (K_m_ × I)/(K_m_ + S) × K_i_, where v_i_ and v_0_ represent the initial rates of hydrolysis of meropenem with or without inhibitor, respectively; I is the concentration of inhibitor; K_i_ is the inhibition constant; K_m_ value is Henri–Michaelis constant, and S is the concentration of the reporter substrate [42,43]. The 50% inhibitory concentration values (*IC*_50_) were determined following the hydrolysis of meropenem at increasing concentrations of each Nb. Each kinetic value represents the average of three independent measurements.

In order to verify the inhibition model, K_m_ and k_cat_ values were calculated using meropenem at increasing concentrations ranging from 50 μM to 250 μM and different concentrations of Nbs (Nb02NDM-1 from 9 nM to 30 nM; Nb12NDM-1 from 400 nM to 700 nM; Nb17NDM-1 from 5 nM to 10 nM).

## 4. Conclusions

In this study, we described the successful strategy to select Nbs with high inhibitory potential toward recombinant NDM-1 enzyme. Nb02NDM-1 and Nb17NDM-1 were shown to be the most potent NDM-1 enzyme inhibitors. Notably, to the best of our knowledge, no studies have reported the inhibitory effect of Nbs on the recombinant NDM-1 enzyme, providing valuable insights for future research in the field of antimicrobial resistance.

## Figures and Tables

**Table 1:** Determination of NDM-1 K_m_ and k_cat_ in presence of different concentrations of Nbs. Each kinetic value is the mean of three different measurements.

MBLs + Nbs	K_m_ (μM)	k_cat_ (s^−1^)
NDM-1	80 ± 2	75 ± 1
NDM-1 + Nb02 (9 nM)	71 ± 3	65 ± 2
NDM-1 + Nb02 (30 nM)	40 ± 1	37 ± 0.5
NDM-1 + Nb12 (400 nM)	65 ± 4	58 ± 2
NDM-1 + Nb12 (700 nM)	34 ± 2	31 ± 1
NDM-1 + Nb17 (5 nM)	53 ± 3	36 ± 0.5
NDM-1 + Nb17 (10 nM)	35 ± 1	21 ± 0.8

**Table 2:** Kinetic parameters of the different inhibitors. Each kinetic value is the mean of three different measurements.

NDM-1	K_i_ (nM)	*IC*_50_ (nM)
Nb02NDM-1	4.7 ± 0.3	26.0 ± 1
Nb12NDM-1	117 ± 8	485 ± 15
Nb17NDM-1	3.7 ± 0.5	7.6 ± 1.2

## Data Availability

Selected Nanobodies can be provided by NanoBioMediKa and DISCAB teams.

## Supplementary Materials

The following supporting information can be downloaded at: https://www.mdpi.com/article/10.3390/molecules29071431/s1, Figure S1: Library construction. DNAs amplified with PCR were observed using agarose gel electrophoresis; Figure S2: Protein expression analysis of purified Nanobodies: Eluted fractions obtained after two steps of purification were concentrated and separated on 16% SDS-PAGE gel under reducing conditions; gel was stained with Coomassie blue; Figure S3: Inhibitory effect of Nb02NDM-1, Nb12NDM-1, and Nb17NDM-1 against the NDM-1 enzyme: (A) the residual activity (%) of NDM-1 using the increased concentration of Nb02NDM-1, (B) the residual activity (%) of NDM-1 using the increased concentration of Nb12NDM-1, and (C) the residual activity (%) of NDM-1 using the increased concentration of Nb17NDM-1.

## Abbreviations

MβLs: metallo-β-lactamases; NDM-1, New Delhi metallo-β-lactamase-1; VIM, verona integron-encoded metallo-β-lactamase; IMP, imipenemase; IgG, immunoglobulin G; HCAbs, heavy-chain homodimer antibodies; Nb, nanobody; and VHH, heavy-chain variable domains.
