# Supplementary material for: Camel-Derived Nanobodies as Potent Inhibitors of New Delhi Metallo-β-Lactamase-1 Enzyme
Source: Molecules. 2024 Mar 22;29(7):1431. doi: 10.3390/molecules29071431 (PMC11013165; doi:10.3390/molecules29071431)
Supplement: Supplementary file 1 [file molecules-29-01431-s001.zip › molecules-2842841-supplementary.pdf]

molecules

MDPI

Supplementary Mater

Camel-Derived Nanobodies as Potent Inhibitors of New Delhi Metallo-β-lactamase-1 Enzyme

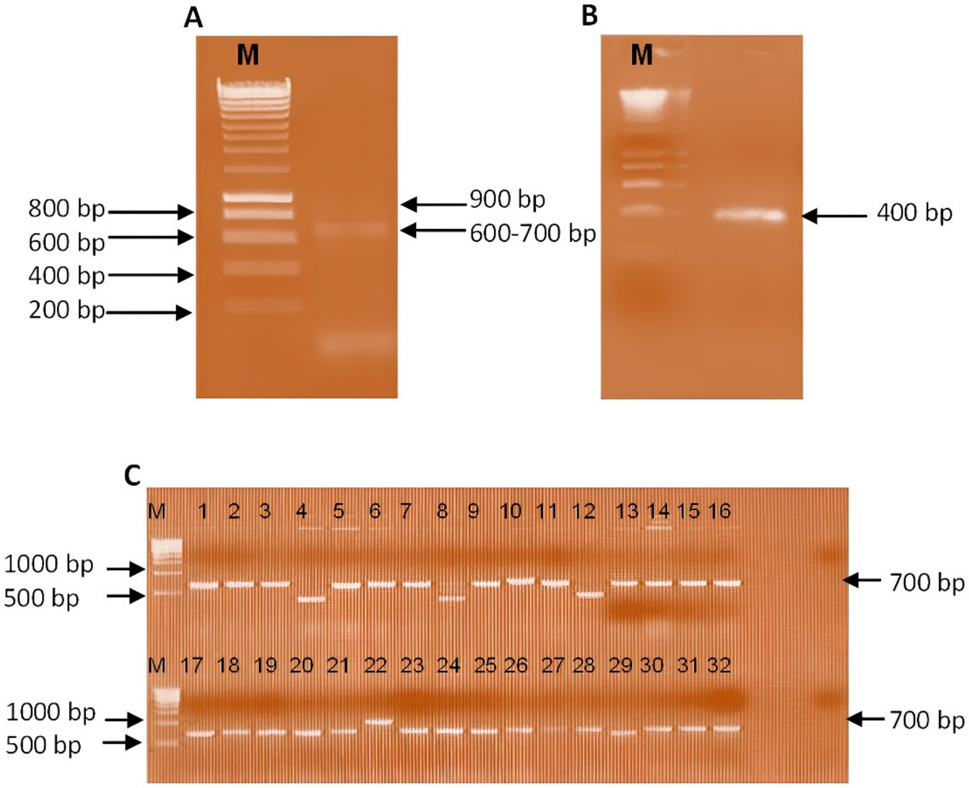

Figure S1. Library construction. DNAs amplified with PCR were observed using agarose gel electrophoresis: **(A)** the first PCR was performed using CALL001/CALL002 primers to amplify the variable domains of heavy-chains IgGs, **(B)** the DNA gene of 700 bp encoding VHH fragments was extracted from agarose gel and amplified with nested PCR using A6E/PMCF primers, and **(C)** the estimation of the correct insertion rate of VHH genes with PCR amplification using MP57/GIII primers.

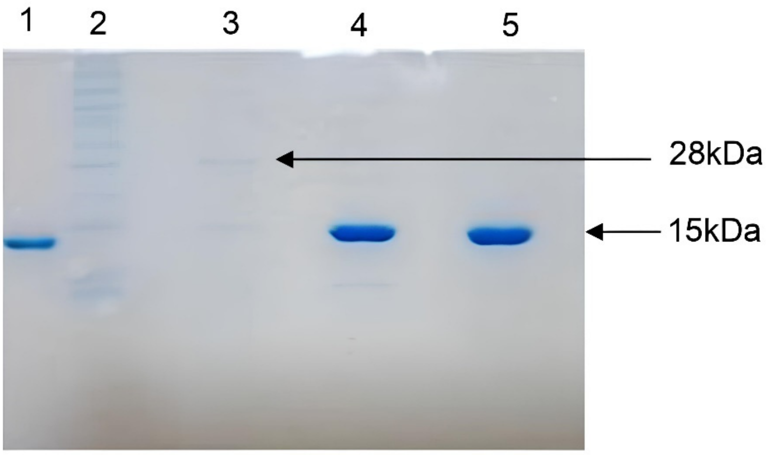

Figure S2. Protein expression analysis of purified Nanobodies: Eluted fractions obtained after two steps of purification were concentrated and separated on 16% SDS-PAGE gel under reducing conditions; gel was stained with Coomassie blue. Lane 1 represents the Nb17NDM-1 fraction; lane 2**:** marker (SHARPMASS™ VII Protein MW marker EPS026500); lane 3**:** periplasmic extract containing Nb17NDM-1; lane 4**:** Nb12NDM-1 fraction; and lane 5**:** Nb02NDM-1 fraction. Nanobodies are visible at 15 kDa, i.e., the molecular weight corresponding to a particular nanobody. The 28 kDa band corresponds to the TEM enzyme, which is endogenously produced by the *WK6* bacterial strain.

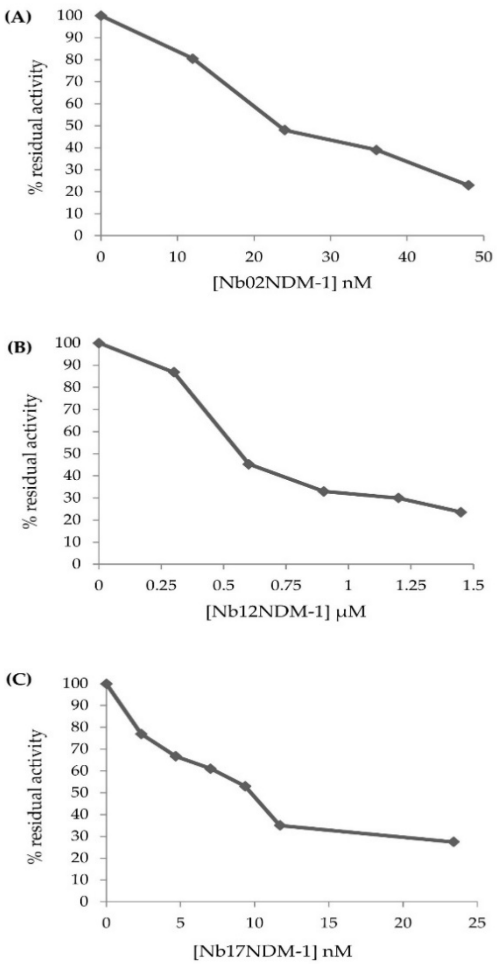

Figure S3. Inhibitory effect of Nb02NDM-1, Nb12NDM-1, and Nb17NDM-1 against the NDM-1 enzyme: (A) the residual activity (%) of NDM-1 using the increased concentration of Nb02NDM-1, (B) the residual activity (%) of NDM-1 using the increased concentration of Nb12NDM-1, and (C) the residual activity (%) of NDM-1 using the increased concentration of Nb17NDM-1. Each kinetic value is the mean of three different measurements; error was below 5%.
